# Serum neurofilament light reflects cognitive dysfunctions in children with obstructive sleep apnea

**DOI:** 10.1186/s12887-022-03514-9

**Published:** 2022-07-26

**Authors:** Yewen Shi, Yani Feng, Xi Chen, Lina Ma, Zine Cao, Lei Shang, Bingjie Zhao, Ningning She, Yitong Zhang, Chao Si, Haiqin Liu, Junjie Zhao, Xiaoyong Ren

**Affiliations:** 1 Department of Otorhinolaryngology Head and Neck Surgery, The Second Affiliated Hospital of Xi’an Jiaotong University, No.157, Xiwu Road, Xi’an, 710004 Shaanxi People’s Republic of China; 2 Department of Health Statistics, School of Public Health, The Fourth Military Medical University, Xi’an, Shaanxi China

**Keywords:** Neurofilament proteins, Tau proteins, Cognitive dysfunctions, Obstructive sleep apnea, Children

## Abstract

**Background:**

In children, obstructive sleep apnea (OSA) can cause cognitive dysfunctions. Amyloid-beta and tau are elevated in OSA. Neurofilament light (NfL) is a marker of neuro-axonal damage, but there are no reports of NfL for OSA. The objective was to investigate the serum levels of NfL and tau in children with or without OSA and explore their relationship with cognitive dysfunctions caused by OSA.

**Methods:**

This retrospective case–control study included children diagnosed with adenoid tonsil hypertrophy from July 2017 to September 2019 at the Second Affiliated Hospital of Xi’an Jiaotong University. Correlations between cognitive scores and tau and NfL were examined.

**Results:**

Fifty-six OSA and 49 non-OSA children were included. The serum NfL levels were higher in the OSA group (31.68 (27.29–36.07) pg/ml) than in the non-OSA group (19.13 (17.32–20.95) pg/ml) (*P* < 0.001). Moreover, NfL was correlated with the course of the disease, apnea–hypopnea index (AHI), obstructive apnea index (OAI), obstructive apnea–hypopnea index (OAHI), average oxygen saturation (SaO_2_), respiratory arousal index (RAI), and cognitive dysfunctions evaluated by the Chinese Wechsler Intelligence Scale for Children (C-WISC) (all *P* < 0.05). The area under the receiver operating characteristics curve (AUC) of NfL was 0.816 (95%CI: 0.736–0.897). Multiple regression analysis revealed that NfL was significantly associated with verbal intelligence quotient (VIQ), performance intelligence quotient (PIQ) and full-scale intelligence quotient (FIQ) (*P* < 0.001, respectively).

**Conclusions:**

Serum NfL levels are associated with the severity of cognitive dysfunctions in children diagnosed with adenoid tonsil hypertrophy and might be a candidate noninvasive, objective marker to identify cognitive dysfunctions in children with OSA.

## Background

Obstructive sleep apnea (OSA) is a common sleep disorder caused by repetitive episodes of upper airway obstruction due to an obstruction or increased airway resistance [1], with an incidence rate of 2%-3% in children [2], 33.9% in men, and 17.4% in women [3]. The risk factors of OSA include adenoid tonsil hypertrophy (especially in children), obesity, race, craniofacial deformity, and neuromuscular diseases [4]. In addition, OSA can lead to complications like cognitive dysfunctions, excessive daytime sleepiness, nightly snoring episodes, apneas, choking, increased effort to breathe, hyperextension of the neck, restless sleep, night sweats, nocturnal enuresis, and parasomnias [5, 6]. These complications in children, especially cognitive dysfunctions have arisen widespread worries and anxieties among parents [6] and attracted extensive research recently.

Cognitive dysfunctions, including irritability, impaired attention, emotional instability, decreased intelligence, and learning disabilities, is a major complication of OSA in children and can usually be measured with tests such as the Wechsler Adult Intelligence Scale-Revised, the Psychomotor Vigilance Task, and the Steer Clear Performance Test [7]. Zhao et al. [8] used the China-Wechsler Younger Children Scale of Intelligence (C-WYCSI) and China-Wechsler Intelligence Scale (C-WISC) for Children and found that mild to moderate OSA was associated with an increased risk of cognitive dysfunctions in children, especially in those < 6 years old. It has been shown that treatment of OSA results in improvements in attention and likely improvements in cognitive abilities [9–11]. Cognitive dysfunctions in children with OSA are attributed to neuronal structural abnormality and dysfunction, such as abnormal neural activities in some brain regions [12, 13], neuronal injury in the hippocampus and frontal cortex [14], regional grey matter reduction [15], brain white matter integrity impairment [16] and altered regional brain cortical thickness [17], damage caused by intermittent hypoxia, and hypercapnia in certain brain regions [10, 18]. In addition, these neuronal changes were always more pronounced in children with moderate-severe OSA. Therefore, early diagnosis and treatment of pediatric OSA are very important. There exists subjective error due to the individual differences in children’s compliance and examiners in these scales. As a result, we need a more objective and convenient measure of neurocognitive function to enable risk and vulnerability stratification during the initial evaluation of snoring and to assess cognitive dysfunction in children with OSA.

As a neuroprotective biomarker, insulin-like growth factors 1 (IGF-1) levels were higher in children with OSA and no neurocognitive deficits. Besides, IGF-1 levels were lower in children with OSA and cognitive deficits [19]. As for biomarkers of Alzheimer's disease, Amyloid β protein 42 (Aβ42) and pre-senilin 1 (PS1) levels were found to increase in pediatric OSA and declined after treatment of OSA with adenotonsillectomy [20].

Recently, some biomarkers of brain injury have been detected in adults with OSA. In a study of 119 male Vietnam War veterans, Elias et al. [21] found that Amyloid β (Aβ) was higher in the OSA group than in the control group quantified by the standardized uptake value ratio of ^18^F-florbetaben. Aβ40 and Aβ42 levels in cerebrospinal fluid (CSF) were found to be lower in OSA patients than in controls, and the apnea–hypopnea index (AHI) was correlated to the Aβ42/Aβ40 ratio [22]. Total tau concentrations in plasma were elevated in young participants with moderate-severe OSA than in young participants with mild or no OSA [23].

Neurofilament light (NfL) is a marker of neuro-axonal damage and can be measured in CSF and serum [24]. As an important marker of brain injury, NfL has been tested and proposed as a promising biomarker of neuroaxonal injury in various neurodegenerative diseases, such as traumatic brain injury [25], subarachnoid hemorrhage [26], sports-related injury [27], military injury [28], and ageing-related changes [24]. Still, the NfL level in children with OSA has not been reported yet.

Therefore, this study aimed to investigate the serum levels of NfL and tau in children with or without OSA and explore their relationship with cognitive dysfunctions potentially caused by OSA. The results of this study could suggest objective blood diagnostic biomarkers of cognitive dysfunctions in children with OSA.

## Methods

### Study design and patients

This retrospective case–control study included children diagnosed with adenotonsillar hypertrophy from July 2017 to September 2019 at the Department of Otolaryngology-Head and Neck Surgery of the Second Affiliated Hospital of Xi’an Jiaotong University.

The inclusion criteria were 1) ≤ 14 years of age, 2) diagnosed with adenoid tonsil hypertrophy [29, 30], 3) underwent polysomnography (PSG), C-WISC, and blood examinations, and 4) complete records. The exclusion criteria were 1) prematurity, 2) any genetic syndrome associated with cognitive disabilities or any chronic or psychiatric condition, 3) use of psychotropic or sedative medicine affecting memory or sleep, 4) neurological abnormalities according to medical history, radiological examination, or electroencephalogram, 5) medical history of visual or hearing disorder, 6) life-threatening sleep apnea, 7) other sleep problems such as insomnia, parasomnia, bedtime behavior difficulties, restless legs syndrome, or abnormal movement during sleep, or 8) any prior treatment for snoring, sleep disordered breathing, or OSA.

The procedures used in this study adhered to the tenets of the Declaration of Helsinki. The study was approved by the Ethics Committee of the Second Affiliated Hospital of Xi’an Jiaotong University (approval no. 2017058). The requirement for individual informed consent was waived by the committee because of the retrospective nature of the study.

### Data collection and definition

Age, course of snoring, course of mouth breathing, course of choking, father’s education level, maternal education level, and body mass index (BMI) were recorded. The tonsils were graded according to the criteria of Brodsky [29]: Grade 0, tonsils restricted into the tonsil fossae; Grade 1, tonsils sitting just outside of the tonsillar fossa and occupying ≤ 25% of the area between the tonsillar pillars; Grade 2, tonsils seen in the airway and occupying 25%-50% of the area between the pillars; Grade 3, tonsils occupying 50%-75% of the area between the pillars; and grade 4, tonsils occupying > 75% of the area between the pillars. Adenoids were measured according to published criteria [30]. The adenoidal/nasopharyngeal (A/N) ratio was measured on lateral cephalometric radiographs. The A parameter was the distance from the point of maximal convexity to the line drawn along the straight region of the anterior basioccipital margin. The N parameter was the distance between the posterior superior edge of the hard palate and the anterior inferior edge of the sphenobasioccipital synchondrosis. Children with A/N ≥ 0.7 were diagnosed with adenoidal hypertrophy.

The results of PSG were collected, including AHI, obstructive apnea index (OAI), obstructive apnea–hypopnea index (OAHI), average oxygen saturation (SaO_2_), and respiratory arousal index (RAI). AHI was defined as the number of apneas and hypopneas per hour of total sleeping time. OAI was defined as the number of obstructive apnea events per hour of total sleep time. OAHI was defined as the total number of obstructive apneas, mixed apneas and obstructive hypopneas per hour of total sleep time. OSA diagnosis was based on OAHI ≥ 1 event per hour during PSG [31, 32]. Children who did not satisfy the OSA criteria were assigned to the non-OSA group. PSG was assessed by two sleep specialists.

The children’s cognitive functions were assessed by the C-WISC [8]. The Verbal Intelligence Quotient (VIQ), Performance Intelligence Quotient (PIQ), and Full-Scale Intelligence Quotient (FIQ) were recorded. In addition, serum NfL and total tau levels were recorded.

Fasting blood samples were drawn by venipuncture in the morning immediately after polysomnographic testing, and plasma samples were processed within 60 min (centrifuge for 15 min at 2500 RPM) and stored at − 80 °C until batch assays were undertaken. NfL and total tau concentrations in serum were measured by commercially ELISA assay (detection range: 6—1000 pg/ml) and single-molecule array (detection range: 0–400 pg/mL). Each reaction was performed in triplicate.

### Statistical analysis

The data were analyzed using SPSS 21.0 (IBM SPSS Statistics, RRID: SCR_019096), R language (www.r-project.org) and GraphPad Prism 7 (GraphPad Prism, RRID: SCR_002798). The distribution of the continuous data was tested using the Kolmogorov–Smirnov test. The continuous variables with a normal distribution were presented as means ± standard deviation and analyzed using Student’s t-test. The continuous variables with a skewed distribution were presented as medians (quantiles) and analyzed using non-parametric tests. Categorical variables were presented as n (%) and analyzed using the chi-square test. Spearman’s correlation analysis was performed to assess the associations between biomarkers serum concentrations (NfL and tau) and parameters of PSG and cognitive dysfunctions (VIQ, PIQ and FIQ). The diagnostic value of NfL was assessed using receiver operating characteristic (ROC). The least absolute shrinkage and selection operator (LASSO) regression analyses were used to filter independent variables and avoid multicollinearity [33]. Multiple regression analyses weighted least-square were performed to determine the factors independently influencing VIQ, PIQ and FIQ. *P*-values < 0.05 were considered statistically significant.

## Results

### Characteristics of the patients

**Fig. 1:**
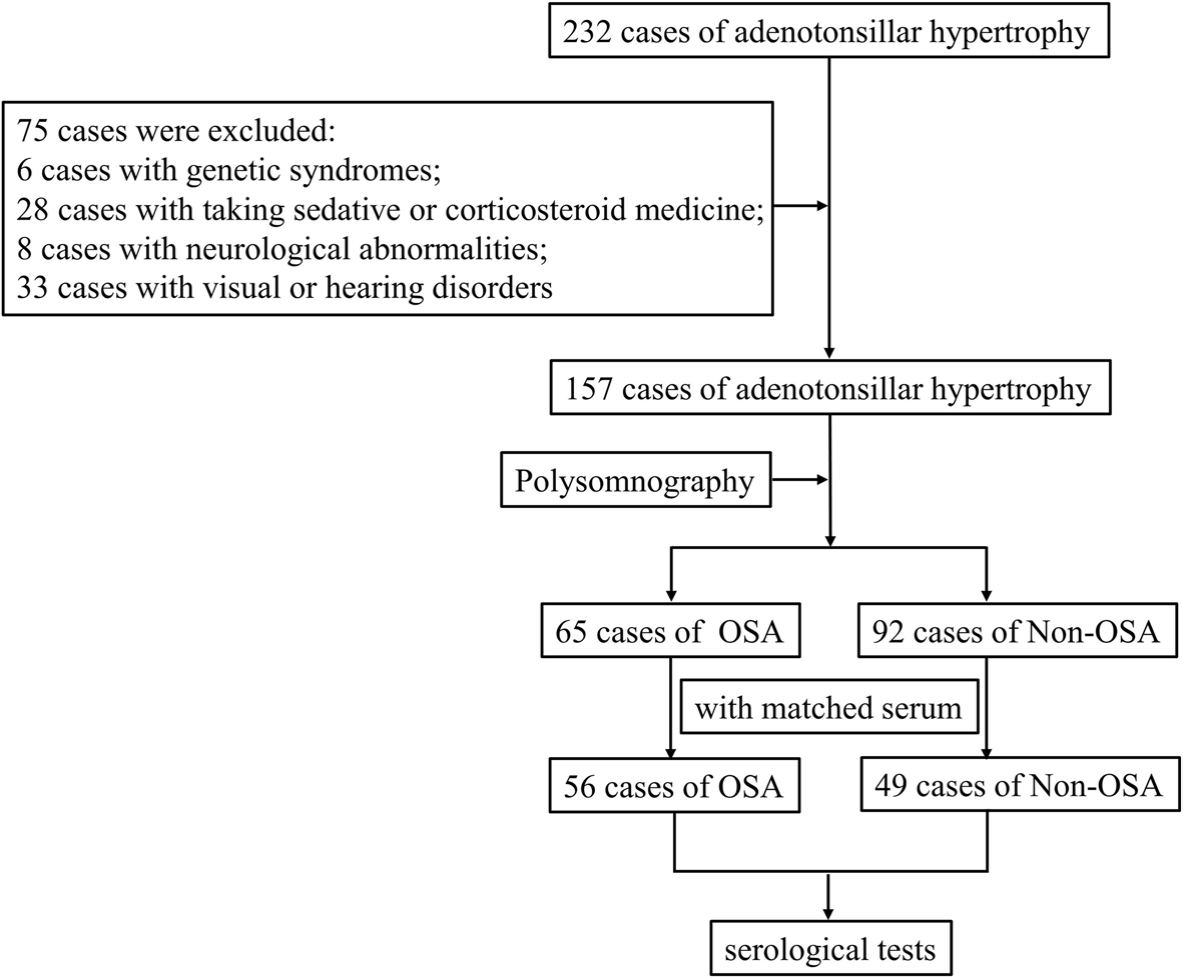
Summary of patient inclusion. Two hundred thirty-two children were diagnosed with adenotonsillar hypertrophy, and 75 children were excluded: 6 with genetic syndromes associated with cognitive disabilities and psychiatric conditions, 28 cases with a history of sedatives or corticosteroids, 8 with neurological abnormalities such as traumatic brain injury and cerebral palsy, and 33 with visual or hearing disorders. The 157 included children all underwent polysomnography (PSG), and 65 were diagnosed with obstructive sleep apnea (OSA). After checking the available serum, 56 OSA and 49 non-OSA children were included in the final analysis

From July 2017 to September 2019, 232 children were diagnosed with adenotonsillar hypertrophy, and 75 children were excluded: six with genetic syndromes associated with cognitive disabilities and psychiatric conditions, 28 cases with a history of sedatives or corticosteroids, eight with neurological abnormalities such as traumatic brain injury and cerebral palsy, and 33 with visual or hearing disorders. The 157 included children all underwent PSG, and 65 were diagnosed with OSA. After checking the available serum, 56 OSA and 49 non-OSA children were included in the final analysis (Fig. 1).

The characteristics of the patients are shown in Table 1. There were no significant differences in sex, age, father’s education level, maternal education level, and BMI between the two groups. Compared with the non-OSA group, the children in the OSA group had longer courses of snoring, mouth breathing, and choking (*P* < 0.001, *P* = 0.001, and *P* < 0.001, respectively). The children in the OSA group had a larger A/N ratio and tonsil size than the non-OSA group (*P* < 0.001 and *P* < 0.001). The AHI, OAI, OAHI, and RAI in the OSA group were significantly elevated compared with the non-OSA group (all *P* < 0.001). SaO_2_ in the OSA group was lower compared with the non-OSA group (*P* = 0.001). VIQ and PIQ in the OSA group were lower than in the non-OSA group (*P* = 0.022 and *P* = 0.016).

**Table 1:** Demographic and clinical characteristics of the patients

	**All**	**OSA**	**Non-OSA**	**P**
**Sex (male/female, n)**	105 (65/40)	56 (27/15)	49 (28/13)	0.347
**Age (year)**	7 (6–7)	7(6–7)	7 (6–8)	0.882
**Course of snoring (months)**	19 (16–22)	27(22–31)	10 (8–13)	< 0.001^**^
**Course of mouth breathing (months)**	15 (12–18)	19 (14–23)	10 (7–13)	0.001^**^
**Course of choking (months)**	8 (6–11)	13 (9–18)	2 (1–3)	< 0.001^**^
**Father’s education level (n)**				0.241
1	4	3	1	
2	7	3	4	
3	31	20	11	
4	30	14	16	
5	32	16	16	
6	1	0	1	
**Maternal education level (n)**				0.586
1	4	3	1	
2	9	4	5	
3	30	19	11	
4	31	13	18	
5	26	14	12	
6	5	3	2	
**BMI (kg/m**^**2**^**)**	17.2 (16.5–17.8)	17.4 (16.5–18.2)	16.9 (15.9–17.9)	0.266
**A/N ratio**	0.67 ± 0.10	0.70 ± 0.10	0.62 ± 0.08	< 0.001^**^
**Tonsil size (n)**				< 0.001^**^
1	19	5	14	
2	48	23	25	
3	38	28	10	
**AHI**	4.9 (2.9–6.8)	8.0 (4.5–11.4)	1.3 (1.2–1.6)	< 0.001^**^
**OAI**	3.6 (1.7–5.5)	3.4 (0.8–5.9)	0.2 (0.1–0.3)	< 0.001^**^
**OAHI**	1.9 (0.5–3.2)	6.3 (2.9–9.8)	0.4 (0.3–0.4)	< 0.001^**^
**Minimum SaO**_**2**_	88.7 (87.8–89.6)	87.5 (86.0–88.9)	90.1 (89.4–90.8)	0.001^**^
**RAI**	0.97 (0.51–1.4)	1.70 (0.87–2.54)	0.13 (0.10–0.17)	< 0.001
**VIQ**	101.21 ± 12.21	98.68 ± 12.87	104.10 ± 10.81	0.022^*^
**PIQ**	99.00 ± 11.90	96.41 ± 12.52	101.96 ± 10.51	0.016^*^
**FIQ**	96.92 ± 13.13	94.95 ± 13.70	99.18 ± 12.19	0.099

The education level:1: completed primary school; 2: completed part secondary education; 3: completed secondary education; 4: completed postsecondary training; 5: completed an undergraduate university degree; 6: completed a postgraduate university degree. *BMI* Body mass index, *A/N* Adenoidal hypertrophy/ nasopharyngeal cavity, *AHI* Apnea–hypopnea index, *OAI* Obstructive apnea index, *OAHI* The obstructive apnea–hypopnea index, *SaO*_2_ Arterial oxygen saturation, *RAI* Respiratory arousal index, *VIQ* Verbal IQ, *PIQ* Performance IQ, *FIQ* Full-Scale IQ. Tonsil size: Grade 0, tonsils restricted into the tonsil fossae; Grade 1, tonsils sitting just outside of the tonsillar fossa and occupying ≤ 25% of the area between the tonsillar pillars; Grade 2, tonsils seen in the airway and occupying 25-50% of the area between the pillars; Grade 3, tonsils occupying 50-75% of the area between the pillars; and grade 4, tonsils occupying > 75% of the area between the pillars. **P < 0.01, *P < 0.05

### Serum NfL and tau

**Fig. 2:**
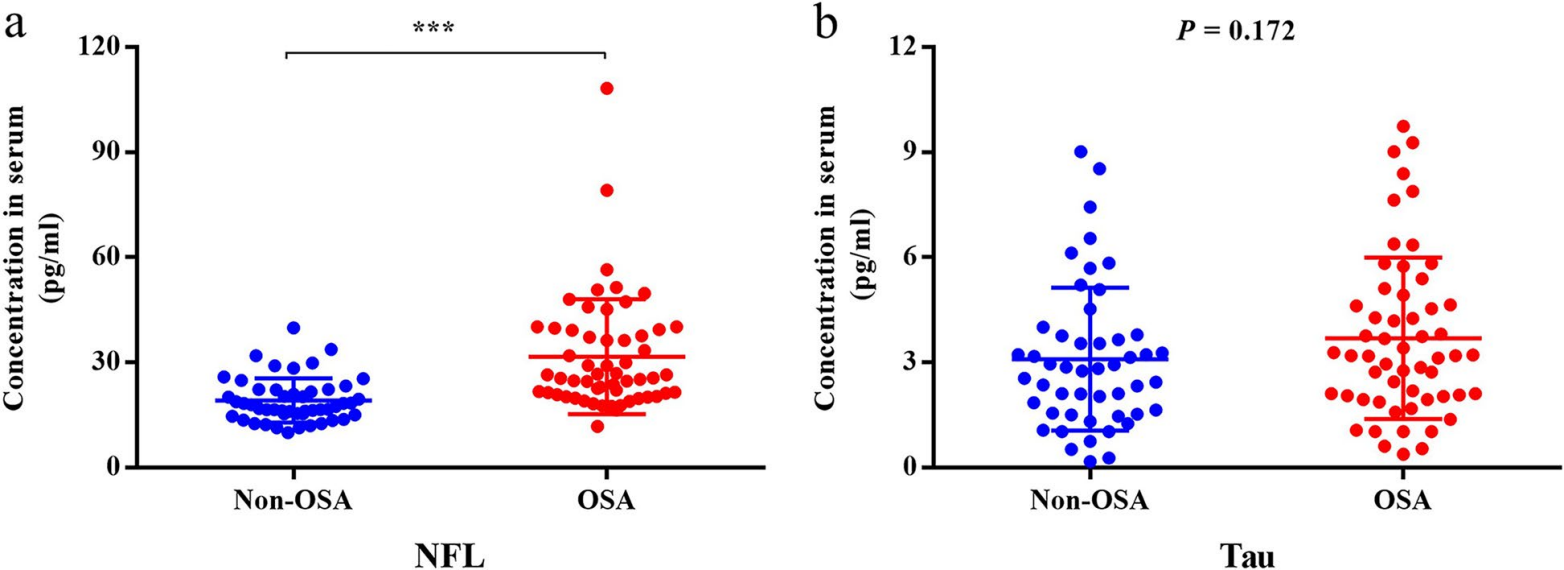
Comparison of serum neurofilament light (NfL) and tau levels between the non-OSA and OSA groups. **a**) The serum NfL levels were high in the OSA group (31.68 (27.29–36.07) pg/ml) compared with the non-OSA group (19.13 (17.32–20.95) pg/ml) (*P* < 0.001). **b**) There were no significant differences in serum tau levels between the two groups (*P* = 0.172)

The serum NfL levels were high in the OSA group (31.68 (27.29–36.07) pg/ml) compared with the non-OSA group (19.13 (17.32–20.95) pg/ml) (*P* < 0.001) (Fig. 2). There were no significant differences in serum tau levels between the two groups (*P* = 0.172).

### Correlations between NfL, tau and clinical parameters

**Fig. 3:**
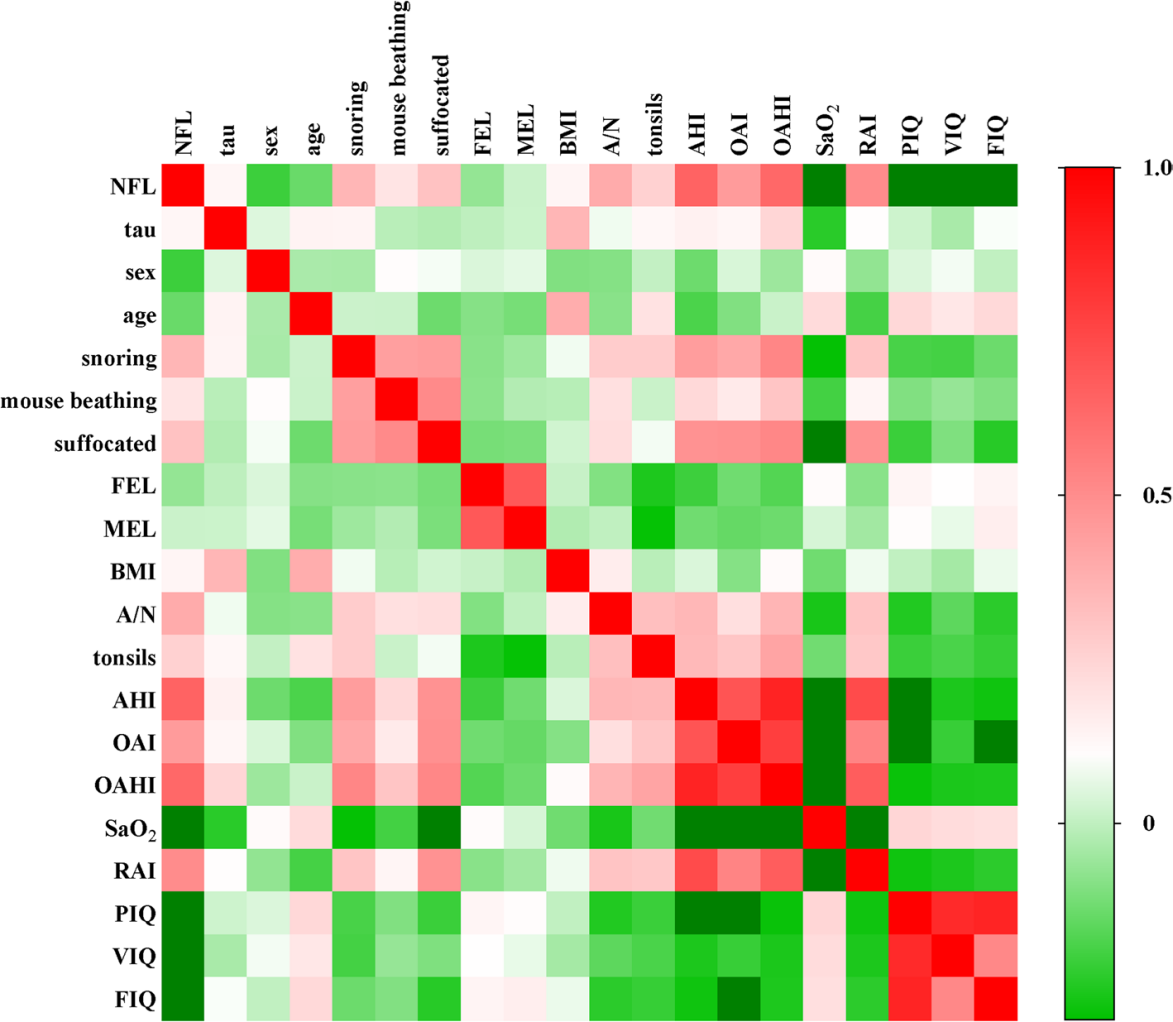
Multivariate correlations with neurofilament light (NfL), tau and clinical parameters. The numerical value of each cell is the spearman correlation coefficient. The baseline (r) value (white) is 0, the maximum value (red) is 1, the light green is -0.3, and the dark green is below -0.3. **P < 0.01, *P < 0.05

The multivariate correlations analyses proved that NfL, not tau, had a good relationship with some parameters of PSG and C-WISC (Fig. 3). It also revealed that serum NfL levels were correlated with course of snoring, mouth opening, and choking (*P* < 0.001, *P* = 0.049 and *P* = 0.001, respectively), A/N ratio (*P* < 0.001), tonsils size (*P* = 0.007), AHI (*P* < 0.001), OAI (*P* < 0.001), OAHI (*P* < 0.001), SaO_2_ (*P* < 0.001), RAI (*P* < 0.001), VIQ (*P* < 0.001), PIQ (*P* < 0.001) and FIQ (*P* < 0.001) (Table 2).

**Table 2:** Correlation analysis between serum neurofilament light (NfL) levels and other parameters

**Variables**	**NfL levels**	
	**Spearman correlation**	**P**
**Course of snoring**	0.354	< 0.001^***^
**Course of mouth breathing**	0.193	0.049^*^
**Course of choking**	0.313	0.001^**^
**A/N**	0.395	< 0.001^***^
**Tonsil size**	0.260	0.007^**^
**AHI**	0.647	< 0.001^***^
**OAI**	0.452	< 0.001^***^
**OAHI**	0.631	< 0.001^***^
**Minimum SaO**_**2**_	-0.430	< 0.001^***^
**RAI**	0.504	< 0.001^***^
**VIQ**	-0.447	< 0.001^***^
**PIQ**	-0.480	< 0.001^***^
**FIQ**	-0.388	< 0.001^***^

*A/N* Adenoidal hypertrophy/nasopharyngeal cavity, *AHI* Apnea–hypopnea index, *OAI* Obstructive apnea index, *OAHI* The obstructive apnea–hypopnea index, *SaO*_2_ Arterial oxygen saturation, *RAI* Respiratory arousal index, *VIQ* Verbal IQ, *PIQ* Performance IQ, *FIQ* Full-Scale IQ. Tonsil size: Grade 0, tonsils restricted into the tonsil fossae; Grade 1, tonsils sitting just outside of the tonsillar fossa and occupying ≤ 25% of the area between the tonsillar pillars; Grade 2, tonsils seen in the airway and occupying 25-50% of the area between the pillars; Grade 3, tonsils occupying 50%-75% of the area between the pillars; and grade 4, tonsils occupying > 75% of the area between the pillars. ***P < 0.001, **P < 0.01, *P < 0.05

### Diagnostic performance of NfL

**Fig. 4:**
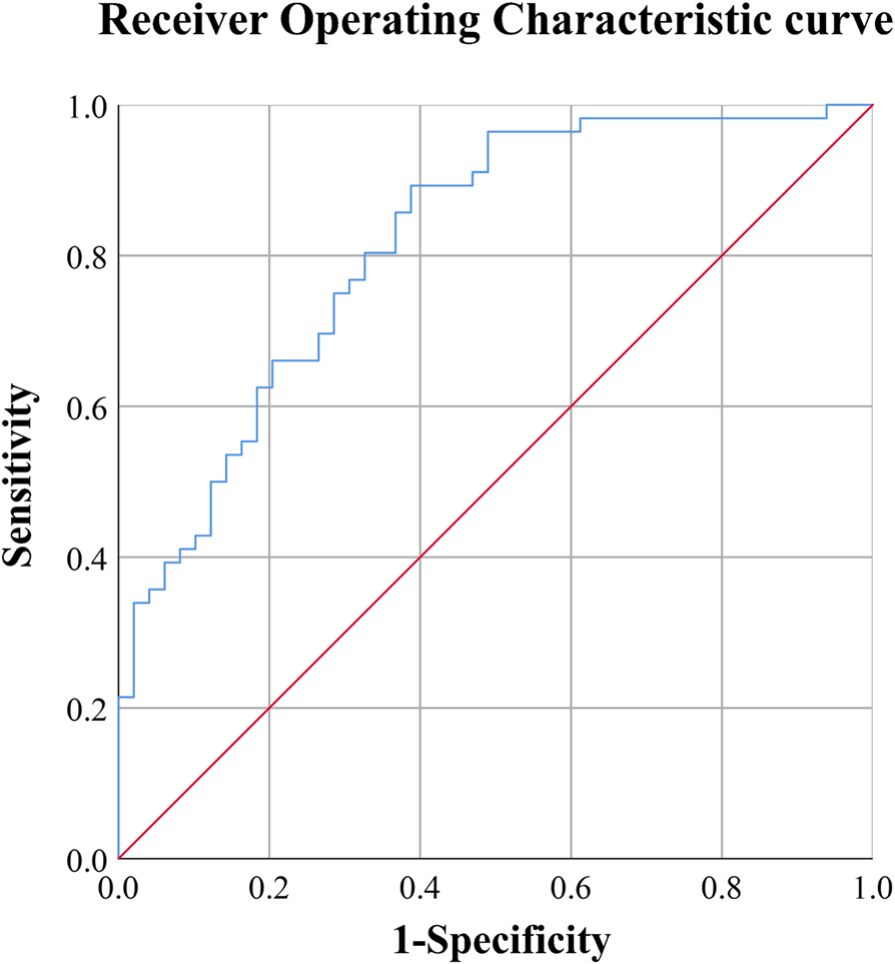
Diagnostic performance of neurofilament light (NfL). Area under the curve (AUC) = 0.816 (95% confidence interval: 0.736–0.897), *P* < 0.001

Serum NfL levels were able to discriminate between OSA and non-OSA children with an AUC of 0.816 (95%CI: 0.736–0.897) (*P* < 0.001) and a maximal Youden index of 0.505 (Fig. 4). Using a cutoff value of NfL of 18.75 pg/ml resulted in 89.3% sensitivity and 61.2% specificity (Table 3).

**Table 3:** Diagnostic performance of neurofilament light (NfL)

**Cutoff (pg/ml)**	**Sensitivity**	**Specificity**	**Youden index**
18.875	0.893	0.612	0.505
19.636	0.857	0.633	0.490
18.973	0.875	0.612	0.487
18.618	0.893	0.592	0.485

###  Multiple regression analyses

**Fig. 5:**
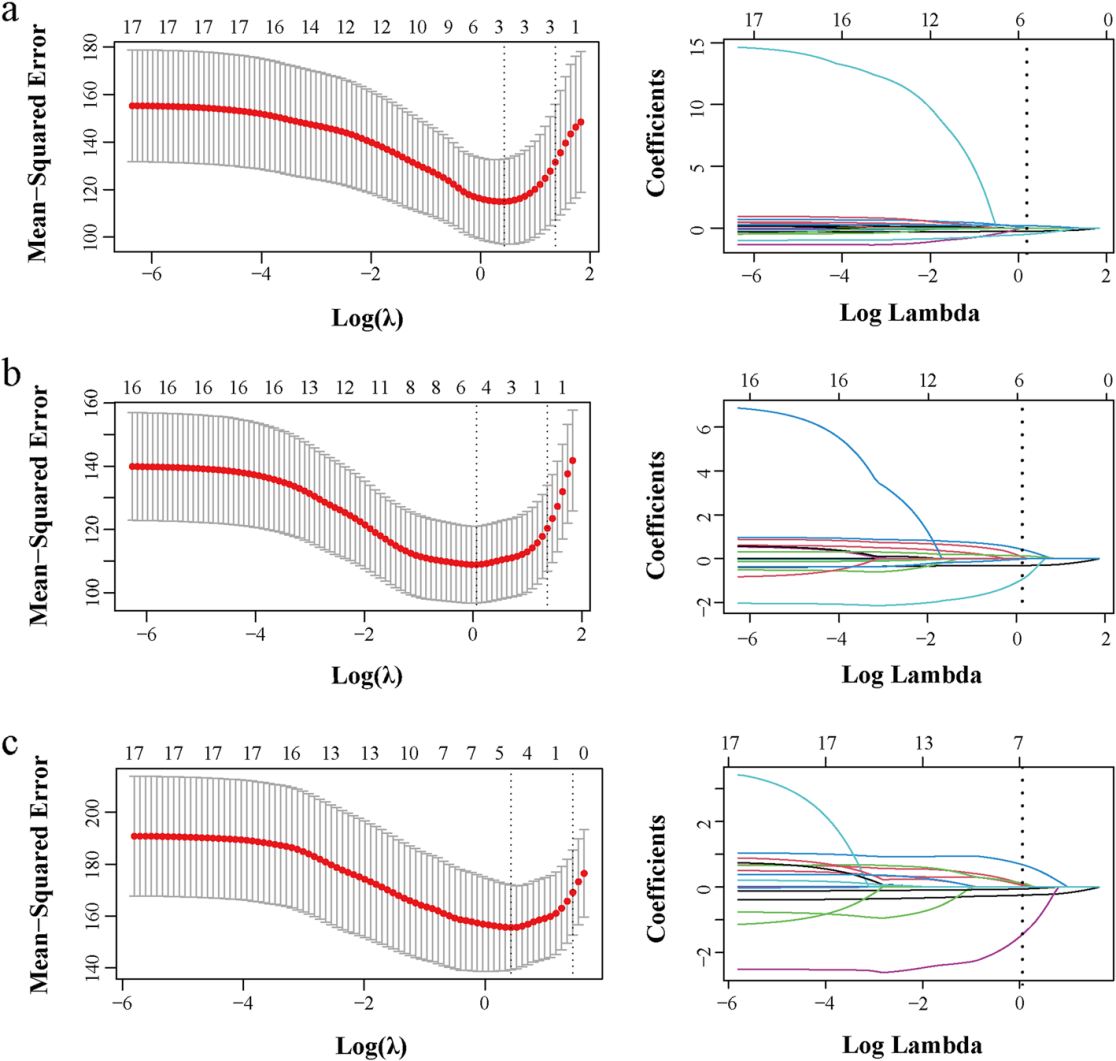
Least absolute shrinkage and selection operator (LASSO) regression analyses for VIQ (**a**), PIQ (**b**) and FIQ (**c**). The left panels show the selection of the optimal parameters (lambda) in the LASSO model used the minimum criterion of tenfold cross-validation. Partial likelihood deviation curves were plotted by lambda. Two dotted vertical lines were drawn at the optimal values at the minimum criterion (right) and the 1-SE criterion (left). The right panels show that LASSO coefficient profiles of 17 features. A coefficient profile plot was produced against the log lambda sequence. Vertical lines were drawn at the value selected using tenfold cross-validation, where optimal lambda resulted from the features with non-zero coefficients

To avoid the multicollinearity of the data, LASSO regression analyses were used to filter independent variables. The selection of the optimal parameters (lambda) in the LASSO model used the minimum criterion of tenfold cross-validation. Partial likelihood deviation curves were plotted by lambda. Two dotted vertical lines were drawn at the optimal values at the minimum criterion and the 1-SE criterion. LASSO coefficient profiles of 17 features (Nfl level, tau level, sex, age (year), course of snoring (months), course of mouth breathing (months), course of choking (months), father’s education level, maternal education level, BMI (kg/m2), A/N ratio, tonsil size, AHI, OAI, OAHI, minimum SaO_2_ and RAI), VIQ, PIQ, and FIQ were separately analyzed as dependent variables (Fig. 5).

Multiple regression analyses weighted least-square were performed among the features with *P*-values lass 0.05 in LASSO models. It revealed that VIQ, PIQ and FIQ were significantly associated with NfL (*P* < 0.001, respectively). Furthermore, VIQ and PIQ were also significantly associated with minimum SaO_2_ (*P* < 0.001, respectively) and RAI (*P* < 0.001, respectively) (Table 4).

**Table 4:** Multiple regression analysis with VIQ, PIQ, FIQ

		**Estimate**	**Std. Error**	**t value**	**Pr( >|t|)**	
**VIQ**	**NfL**	-0.31557	0.01130	-27.922	< 0.001^***^	*P* < 0.001
	**Minimum SaO**_**2**_	0.24777	0.04707	5.264	< 0.001^***^	Adjusted *R*^2^ = 0.9602
	**RAI**	-0.89795	0.18802	-4.776	< 0.001^***^	*F* = 836.6
**PIQ**	**NfL**	-0.35853	0.02625	-13.658	< 0.001^***^	*P* < 0.001
	**Age**	1.04610	0.09327	11.216	< 0.001^***^	Adjusted R^2^ = 0.9216
	**Father’s education level**	0.85349	0.12742	6.698	< 0.001^***^	*F* = 204.6
	**Tonsil size**	-1.69374	0.23067	-7.343	< 0.001^***^	
	**Minimum SaO**_**2**_	0.03552	0.09932	0.358	0.721	
	**RAI**	-0.45600	0.16713	-2.728	< 0.01^**^	
**FIQ**	**NfL**	-0.25107	0.02531	-9.921	< 0.001^***^	*P* < 0.001
	**Age**	1.18906	0.06821	17.432	< 0.001^***^	Adjusted *R*^2^ = 0.9769
	**Course of choking**	-0.07922	0.02250	-3.520	< 0.001^***^	*F* = 879.2
	**Maternal education level**	0.84824	0.22024	3.851	< 0.001^***^	
	**Tonsil size**	-2.59813	0.39786	-7.063	< 0.001^***^	

*Nfl* Neurofilament light, *SaO*_*2*_ Arterial oxygen saturation, *RAI* Respiratory arousal index, *VIQ* Verbal IQ, *PIQ* Performance IQ, *FIQ* Full-Scale IQ. The education level:1: completed primary school; 2: completed part secondary education; 3: completed secondary education; 4: completed postsecondary training; 5: completed an undergraduate university degree; 6: completed a postgraduate university degree. Tonsil size: Grade 0, tonsils restricted into the tonsil fossae; Grade 1, tonsils sitting just outside of the tonsillar fossa and occupying ≤ 25% of the area between the tonsillar pillars; Grade 2, tonsils seen in the airway and occupying 25–50% of the area between the pillars; Grade 3, tonsils occupying 50–75% of the area between the pillars; and grade 4, tonsils occupying > 75% of the area between the pillars. ***P < 0.001, **P < 0.01, *P < 0.05

## Discussion

OSA in children can potentially cause cognitive dysfunctions [9–11], but the available cognition assessment tools exist subjective measurement error. NfL is a marker of neuro-axonal damage [24–28], but there are no reports of NfL for OSA. This study aimed to investigate the serum levels of NfL and tau in children with or without OSA and explore their relationship with cognitive dysfunctions potentially caused by OSA. Children with OSA had elevated serum levels of NfL compared with children diagnosed with adenotonsillar hypertrophy but without OSA. NfL correlated with PSG (AHI, OAI, OAHI, SaO_2_, and RAI) and C-WISC (VIQ, PIQ and FIQ) parameters. Since the eligibility criteria excluded the influence from comorbidities such as the genetic syndromes, history of sedatives and, neurological abnormalities, the most likely etiology of increased concentrations of NfL was OSA. Therefore, the results suggest that serum NfL levels are associated with the severity of cognitive dysfunctions in children diagnosed with adenotonsillar hypertrophy and might be a candidate noninvasive, objective marker to identify cognitive dysfunctions in children with OSA.

Recently, some other approaches were used to assess OSA with cognitive dysfunctions such as imaging and serologic examinations [4]. Some researchers used magnetic resonance imaging (MRI) to assess brain structural and functional changes in OSA. The cerebral metabolic rate of oxygen was found to be decreased in OSA patients compared with controls in response to apnea [34]. In addition, OSA patients had significantly altered functional connectivity in multiple brain regions, showing less efficient integration and declined regional topological properties and specialization characteristics [35]. Based on MRI, cognitive dysfunctions in children with OSA were reported to be associated with neuronal structural abnormality and dysfunction, such as abnormal neural activities in some brain regions [12, 13], neuronal injury in the hippocampus and frontal cortex [14], regional grey matter reduction [15], brain white matter integrity impairment [16] and altered regional brain cortical thickness [17]. Serologic examinations also found that Aβ and tau were higher in the OSA group than in the control group in children or adults [20, 21, 23].

NfL, a novel marker of neuronal and axonal injuries, has attracted widespread attention and has been studied in a wide range of neurologic disorders [24, 25, 28]. A study showed that serum NfL levels are stable in people below 60 years but significantly increased in people above 60 years [24]. Moreover, the NfL levels are significantly elevated in traumatic brain injury patients and have a good diagnostic performance for traumatic brain injury, with 92.3% sensitivity and 88.9% specificity [25]. In subarachnoid hemorrhage, plasma NfL levels are associated with disease severity during early stages and with poor 30-day functional outcome and mortality [26]. Some studies showed that NfL provides additional information to help discriminate clinical severity in dementia, particularly as the diagnosis progresses from mild cognitive impairment to Alzheimer’s disease (AD), and NfL starts to increase 10 years before AD diagnosis [36, 37]. Therefore, plasma NfL levels can be regarded as a noninvasive biomarker to identify those at risk for mild cognitive impairment. Serum NfL appears to be an independent determinant of future brain volume loss [24]. Meanwhile, some researchers also found that grey matter volumes were reduced in pediatric obstructive sleep apnea [17, 38]. Macey et al. [17] found significant grey matter volume reduction in OSA throughout the superior frontal and prefrontal areas, superior and lateral parietal cortices, and superior temporal lobe.

In the present study, the serum levels of NfL were measured in children diagnosed with adenotonsillar hypertrophy, and the NfL levels in OSA were increased compared with non-OSA, which strongly suggests that OSA can indeed lead to brain injury. Our results also showed that the NfL levels were correlated with VIQ, PIQ and FIQ, indicating that the NfL levels represented the cognitive status evaluated by the C-WISC. NfL levels might reflect the severity of cognitive dysfunctions in children with OSA. Together with previous reports [14, 17, 39], these results suggested that OSA can cause brain injuries in children to some extent, which may be the cause of cognitive dysfunctions. NfL might be a novel biomarker of cognitive dysfunctions in children with OSA. Interestingly, the results also showed that the NfL levels were also correlated with the courses of snoring, mouth breathing and choking, A/N ratio, tonsils size, PSG parameters, and C-WISC parameters, which indicated that these symptoms and signs might be hazard factors of brain injuries caused by OSA in children.

Chronic intermittent hypoxia, sleep fragmentation, and inflammatory activation are the main pathophysiological mechanisms of OSA. In multiple regression models, VIQ, PIQ and FIQ were significantly associated with NfL. VIQ and PIQ were also significantly associated with minimum SaO_2_ and RAI. It is noteworthy that AHI and OAHI, which are used as the objective indicator for the diagnosis of OSA, were not selected as related features in LASSO models. These analyses show that the cognitive deficits associated with pediatric OSA may be more closely related to the drop in blood oxygen levels and sleep fragmentation, rather than sleep apnea/hypopnea.

PSG is the golden diagnostic golden standard of OSA [31, 32], but the evaluation of cognition in children is complex and arduous. There are some questionnaire tools used for the evaluation of cognitive functions in children, such as Conners’ Parents Rating Scale, Child Behavior Checklist, Differential Ability Scale, Developmental Neuropsychological Assessment to assess neurobehavioral problems [14], WISC-III, WISC-IV [39], Hong Kong WISC, Forward Span of the Spatial Span subtest of the Wechsler Memory Scale to assess working memory tasks and visuospatial sketchpad [8], and the C-WISC [8, 40], but there is no gold standard among them. Besides, there exist subjective errors and they are not sensitive enough for early cognitive dysfunctions. Therefore, a more objective and time-efficient way to evaluate neurocognitive function is required to enable risk and vulnerability stratification to assess cognitive outcomes in children.

Serologic examinations are relatively cheap, fast, and accessible, and the development of new serologic biomarkers is essential for diagnosing OSA. Here, the results showed that the AUC of NfL was 0.816 (0.736–0.897), indicating that NfL had relatively good sensitivity and specificity as an accessible diagnostic and screening marker of cognitive dysfunction in OSA patients.

A few studies have reported increased serum and cerebrospinal fluid levels of tau in patients with OSA compared to control subjects [23, 41], which showed that tau might be an indicator of cognitive dysfunctions in patients with OSA. Nevertheless, in the present study, no significant differences in tau levels were identified between the two groups. It might be due to the shorter average duration of OSA in children than in adults.

It is the first study investigating the association between NfL and pediatric OSA. Considering that this study was a retrospective case–control study and the sample size was relatively small, prospective multicenter randomized controlled trials with a larger sample size are needed to confirm the NfL cutoff levels for the diagnosis of OSA and evaluate whether NfL levels are associated with the severity of OSA in children. This study only included patients formally diagnosed with adenotonsillar hypertrophy and who underwent PSG, but whether NfL could be used for OSA-related cognitive dysfunctions screening in the general pediatric population remains to be determined. Future studies will also be necessary to explore the relationships between serum NfL levels and other cognitive evaluation tests in children diagnosed with adenotonsillar hypertrophy and OSA.

## Conclusions

The serum NfL levels were elevated in children with adenotonsillar hypertrophy and OSA compared with children with adenotonsillar hypertrophy but no OSA. The serum levels of NfL were correlated with VIQ, PIQ and FIQ. NfL levels might reflect the severity of cognitive dysfunctions in children with OSA.

## Data Availability

The datasets generated and/or analysed during the current study are not publicly available due to ongoing research projects but are available from the corresponding author on reasonable request.

## Abbreviations

OSA: Obstructive sleep apnea
NfL: Neurofilament light
AHI: Apnea–hypopnea index
OAI: Obstructive apnea index
OAHI: Obstructive apnea–hypopnea index
SaO_2_: Average oxygen saturation
RAI: Respiratory arousal index
C-WISC: Chinese Wechsler Intelligence Scale for Children
AUC: Area under curve
VIQ: Verbal Intelligence Quotient
PIQ: Performance Intelligence Quotient
FIQ: Full-Scale Intelligence Quotient
C-WYCSI: China-Wechsler Younger Children Scale of Intelligence
IGF-1: Insulin-like growth factors 1
PS1: Pre-senilin 1
Aβ: Amyloid β
CSF: Cerebrospinal fluid
PSG: Polysomnography
BMI: Body mass index
A/N: Adenoidal/nasopharyngeal
RAI: Respiratory arousal index
VIQ: Verbal Intelligence Quotient
PIQ: Performance Intelligence Quotient
FIQ: Full-Scale Intelligence Quotient
ROC: Receiver operating characteristic
LASSO: Least absolute shrinkage and selection operator
MRI: Magnetic resonance imaging
AD: Alzheimer’s disease
